# Co‐Occurrence Patterns Do Not Predict Mutualistic Interactions Between Plant and Butterfly Species

**DOI:** 10.1002/ece3.70498

**Published:** 2024-10-30

**Authors:** Esteban Menares, Hugo Saíz, Noëlle Schenk, Enrique G. de la Riva, Jochen Krauss, Klaus Birkhofer

**Affiliations:** ^1^ Department of Ecology Brandenburg University of Technology Cottbus‐Senftenberg Cottbus Germany; ^2^ Institute of Plant Sciences University of Bern Bern Switzerland; ^3^ Department of Animal Ecology and Tropical Biology University of Würzburg Würzburg Germany

**Keywords:** Biodiversity Exploratories, co‐occurrence analysis, flower visitations, mutualistic interactions, presence/absence data, species associations

## Abstract

Biotic interactions are crucial for determining the structure and dynamics of communities; however, direct measurement of these interactions can be challenging in terms of time and resources, especially when numerous species are involved. Inferring species interactions from species co‐occurrence patterns is increasingly being used; however, recent studies have highlighted some limitations. To our knowledge, no attempt has been made to test the accuracy of the existing methods for detecting mutualistic interactions in terrestrial ecosystems. In this study, we compiled two literature‐based, long‐term datasets of interactions between butterflies and herbaceous plant species in two regions of Germany and compared them with observational abundance and presence/absence data collected within a year in the same regions. We tested how well the species associations generated by three different co‐occurrence analysis methods matched those of empirically measured mutualistic associations using sensitivity and specificity analyses and compared the strength of associations. We also checked whether flower abundance data (instead of plant abundance data) increased the accuracy of the co‐occurrence models and validated our results using empirical flower visitation data. The results revealed that, although all methods exhibited low sensitivity, our implementation of the Relative Interaction Intensity index with pairwise null models performed the best, followed by the probabilistic method and Spearman's rank correlation method. However, empirical data showed a significant number of interactions that were not detected using co‐occurrence methods. Incorporating flower abundance data did not improve sensitivity but enhanced specificity in one region. Further analysis demonstrated incongruence between the predicted co‐occurrence associations and actual interaction strengths, with many pairs exhibiting high interaction strength but low co‐occurrence or vice versa. These findings underscore the complexity of ecological dynamics and highlight the limitations of current co‐occurrence methods for accurately capturing species interactions.

## Graphical abstract

Direct measurement of biotic interactions is time‐ and resource‐intensive, especially in species‐rich communities. As a result, inferring species interactions from co‐occurrence patterns has become more common; however, recent studies have highlighted significant limitations in this approach. In particular, evidence of mutualistic interactions in terrestrial ecosystems remains sparse. To address this gap, we compared three co‐occurrence analysis methods to predict species interactions between plants and butterflies in permanent grasslands. Our results show that all methods performed poorly, with high incongruence between predicted co‐occurrence and actual interaction strengths.

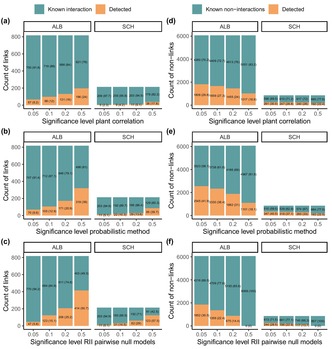

## 1 Introduction

Mutualistic interactions between plants and animals such as pollination are the base that supports many terrestrial ecosystems (Bascompte and Jordano 2013; Mittelbach and McGill 2019). At the community level, interactions may involve dozens or even hundreds of interacting species in complex ways. Therefore, to investigate them, studies should shift from a reductionist, individual‐level to a more systemic, community‐level approach. This is particularly relevant for mutualistic interactions, such as pollination, which are highly relevant not only for ecosystem functioning but also for humans. For instance, pollination is considered a vital ecosystem service valued at an estimated global annual cost of 235–577 million USD (IPBES 2016). Interactions between species are typically observed directly from physical contact between the species involved or, in the case of pollination, quantified from flower visitations (Börschig et al. 2013; Mittelbach and McGill 2019), which is time‐ and resource‐intensive, thereby limiting our ability to extensively study them. Therefore, recently, efforts have been taken to infer species interactions from species co‐occurrence patterns based on incidence or abundance matrices, using statistical methods (Faust and Raes 2012; Lane et al. 2014; Borthagaray, Arim, and Marquet 2014; Harris 2016; Brazeau and Schamp 2019; Thurman et al. 2019).

Pattern analysis involves measuring the frequency of the co‐occurrence of two or more species among different spatial locations and evaluating whether those species show a positive (i.e., co‐occur more often than expected, aggregated) or negative (i.e., co‐occur less often than expected, segregated; Birkhofer, Wolters, and Diekötter 2011; Cazelles et al. 2016) spatial association. A primary concern of co‐occurrence analysis is understanding the extent to which biotic interactions translate into species co‐occurrence patterns (aggregated or segregated), assuming that biotic interactions leave a spatial signal (Goberna and Verdú 2022). Recent studies have tested the capacity of current co‐occurrence methods to infer trophic and non‐trophic (commensalism and facilitation) interactions, including those between marine invertebrates, in rocky intertidal ecosystems (Freilich et al. 2018; Barner et al. 2018), competition in herbaceous plants in grassland ecosystems (Brazeau and Schamp 2019), competition and trophic interactions in amphibians in mountainous ecosystems (Thurman et al. 2019), or plant–plant interactions in a semi‐arid gypsum community (Delalandre and Montesinos‐Navarro 2018). All these studies highlighted, to different degrees, the unreliability of the tested methods as proxies for these ecological interactions, but to our knowledge, none have specifically examined the effectiveness of these methods in detecting mutualistic interactions, such as pollination.

At the community level, mutualistic interactions are normally highly heterogeneous (i.e., the set of interacting species differs across locations) and nested in space (i.e., the species set at one location is a subset of the set at another location), and species interactions are typically weak and asymmetric (i.e., the benefits exchanged between species are not necessarily equal) (Bascompte and Jordano 2013). Regarding the interactions between plants and butterflies, different species of adult lepidopterans have been shown to pollinate herbaceous plants (Andersson et al. 2002) and crops (Rader et al. 2016) in exchange for nectar retribution, in which species from both guilds benefit from each other. Many butterfly species are generalists who visit the flowers of different plant species, but some develop more plant‐specific relationships with only one or two species of plants, such as in Brandenburg (Germany), *Pyrgus alveus* (Hübner, 1803), *Leptidea sinapis* (Linnaeus, 1758), and *Maculinea alcon* (Denis & Schiffermüller, 1775) (Richert and Brauner 2018). In Germany, some states conduct long‐term, annual surveys of butterfly‐plant visitation, which allow these observed interactions to be compared with the associations predicted by co‐occurrence methods. Given the nature of mutualistic interactions, they should be exclusively detected via positive associations obtained from co‐occurrence analysis, and because co‐occurrence analyses tend to better infer positive than negative interactions (Freilich et al. 2018; Araújo and Rozenfeld 2014), we would expect co‐occurrence analyses to perform well when exclusively analyzing mutualistic interactions, such as pollination. Conversely, the low specificity of some butterfly species interactions would reduce the signal strength and hinder the ability of co‐occurrence methods to correctly detect associations, providing an opportunity to test the effectiveness of these methods in this common application scenario. For example, co‐occurrence association analysis has been used to find species interactions for which functional or trophic information is lacking a priori (e.g., Lima‐Mendez et al. 2015). In this study, we tested how well the species associations generated by three different co‐occurrence analysis techniques matched those of empirically measured mutualistic interactions between herbaceous plants and day‐active lepidopteran species in agricultural grassland ecosystems. For this, we compiled two long‐term datasets of butterfly and plant species interactions from literature collected in two regions of Germany, namely, Brandenburg (northeast) and Baden‐Württemberg (southwest), and compared them with observational abundance and presence/absence data collected within a year in the same regions. Given that the extent of the sampling area largely covers the distribution area of the analyzed species (as recommended by Goberna and Verdú 2022), and that co‐occurrence methods are better suited to detect positive interactions (Freilich et al. 2018; Araújo and Rozenfeld 2014), we expected a high level of interaction detection using co‐occurrence methods. We also checked whether using flower abundance data (instead of just plant abundance data) increased the accuracy of the co‐occurrence models and used additional flower visitation data to validate our results. We further checked the strength of the interaction signals in the empirical data by correlating them with the co‐occurrence association strength.

## 2 Materials and Methods

### 2.1 Occurrence Data

To calculate co‐occurrences, we used existing field datasets from BExIS (https://www.bexis.uni‐jena.de/), the information system of the Biodiversity Exploratories (BEs) program (DFG Priority Program 1374), and a large‐scale and long‐term biodiversity research project in three regions of Germany. We focused on two regions: the Swabian Jura (Schwäbische Alb; ALB) in Baden‐Württemberg (southwest) and Schorfheide‐Chorin (SCH) in Brandenburg (northeast), both of which are biosphere reserves. Both regions had a higher proportion of semi‐natural habitats than those typically found in most areas in Germany. The two regions (ALB vs. SCH) have different geological substrates (loess on calcareous bedrock vs. glacial till often covered by glacio‐fluvial or eolian sand), main soil types [Leptosols (in steep slopes) and Cambisols vs. Histosols (drained) and Luvisols], historical management practices [military training area (in parts) vs. intensive agricultural use], and other climatic and environmental parameters, for example, annual rainfall (700–1.000 vs. 480–580 mm) and elevation (460–860 vs. 3–140 m.a.s.l.). Nevertheless, they are managed with similar current management practices and intensities, as described in detail by Fischer et al. (2010)). We selected 89 sites (EPs) in total, 46 in ALB and 43 in SCH, for which we found overlapping field and literature data. Each site was 50 m × 50 m.

The total abundance and richness of butterfly imagines (Lepidoptera: Papilionoidea) were measured during field observations between the beginning of May and mid‐August 2008 at all sites, including three surveys, with 300 m long transects per survey, with each transect comprising six intervals of 50 m (BExIS dataset 12,526; Börschig and Krauss 2016). We pooled the data by first summing the abundances over all intervals, that is, the total abundance per species and survey. Then, the summed abundances were averaged across surveys, that is, the total abundance for the entire year per species and site. Species names and synonyms were checked using Lepiforum (Rennwald and Rodeland n.d.; https://lepiforum.org/) and the Global Lepidoptera Index (Beccaloni et al. 2022). *Leptidea sinapis*, *L. reali*, and *L. juvernica* were considered as *L. sinapis* complex according to Dincă et al. (2011). *Pyrgus alveus*, *P. trebevicensis*, and *P. accrete* were considered as the *P. alveus* complex according to Tshikolovets (2011, pp. 40‐69).

Vegetation abundance was measured at all sites between May and June 2008 as the percentage cover of each species relative to one 4 m × 4 m sampling plot per site (BExIS dataset 23,586; Schäfer 2018). We considered only herbaceous flowering plants and excluded species identified only at the genus level as well as unidentified observations, trees, shrubs, and fern species. Species names and synonyms were checked using the International Plant Names Index (IPNI 2023) and the Plants of the World Online Database (POWO 2023). A table with all butterfly and plant species names and synonyms for both regions can be downloaded from BExIS (dataset 31,733).

### 2.2 Ecological Interactions Data

Data on the interactions between herbaceous flowering plants and lepidopteran imagines were collected from long‐term surveys of flower visitation from the literature for the region ALB (Ebert 2005) and SCH (Richert and Brauner 2018). Data from ALB were collected by the Baden‐Württemberg State Institute for Environmental Protection and the State Museum for Natural History in Karlsruhe, mainly between 1975 and 2005, but also included previously published data. The SCH data covers a period from 1960 to 2018 and includes regional works by different authors, data from the InsectIS Brandenburg database that was evaluated, critically checked, and compared with Gelbrecht et al. (2016, pp. 30‐311), and personal observations of the authors, specially, the work from 2002 to 2005 by F. Gottwald in SCH (Stein‐Bachinger et al. 2010). Interactions were defined as visits per butterfly species to nectar plants regardless of their local abundance or rarity status. We coded the interactions on a categorical scale from zero to one, by adapting the scale of Ebert (2005, p. 273), with 0 representing no interaction (i.e., no flower visits), 0.2 representing either single observation or observation without evaluation, 0.4 representing single observation (but the butterfly exhibits flower constancy), 0.6 representing several times observation, and 0.8 representing frequent to very frequent observation, and 1 representing a strong interaction (i.e., many visits and nectar plant of paramount importance). For region SCH, after discussing with the author of the original work, we excluded the levels 0.4 and 0.8 (Oliver Brauner, personal communication); therefore, the final scale included only 0: no interaction; 0.2: single or few/occasional observation(s), with the butterfly exhibiting flower constancy in some of the observations: 0.6: observed multiple times or frequently; and 1: observed frequently to very frequently or regularly (Richert and Brauner 2018, 194). The value categories in the above lists are minimum values based on multiple observations, so in many cases, greater significance may be possible (Figures S1 and S2). We filtered the interaction dataset only for those species present in the occurrence dataset and calculated the number of interactions; therefore, the total interactions per species did not strictly represent the full range of interactions per species described in the literature. For practical purposes, *Medicago* x *varia Martin* is considered a synonym of *Medicago sativa* aggr. Only in Ebert (2005), both plants were listed, and whenever a butterfly had an interaction with *M*. x *varia Martin* and *M. sativa* aggr., the highest interaction strength was considered. *Valeriana officinalis* included all subspecies and aggregations, and *Vicia sativa* aggr. includes all subspecies of *V. sativa*. that appeared in both books. Also in this case, when a butterfly had an interaction with any of these plants, the highest interaction strength was considered. Initial evaluation of the overlap in species interactions between the two regions revealed that approximately 36% of the interactions were shared between both regions and 64% were exclusively present in one region. Consequently, we conducted separate analyses for the two regions.

### 2.3 Co‐Occurrence Analysis

All analyses were conducted in R version 4.1.2 (R Core Team 2021). To test how well co‐occurrence data between plants and butterflies predicts mutualistic interactions, we used three pairwise methods for species association (co‐occurrence methods), testing against empirical interaction data. We have chosen these because, of the pairwise methods available, they are currently the most widely used and tested in the literature (Barner et al. 2018; Lavender et al. 2019). Of these, two were based on the presence/absence (P/A) data and one on the abundance data. We first filtered the interaction data to include only the species present in the occurrence data. For the P/A data, we transformed the abundance data into Boolean (0/1) and then calculated associations using two different techniques: the RII index (Armas, Ordiales, and Pugnaire 2004) and the probabilistic method proposed by Veech (2013, 2014) using the *cooccur* package in R (Griffith, Veech, and Marsh 2016, pp. 1‐12). The RII is an index used to compare the intensity of plant interactions. RII ranges from −1 to 1, is symmetrical around zero, and has a negative value for competition and a positive value for facilitation but can be calculated for any type of net interaction. RII also exhibits logarithmic behavior, asymptotic to the *y*‐axis lines 1 and − 1 as a sigmoid function. To derive the RII, we first calculated the observed co‐occurrences (i.e., the number of sites with both species present) and the expected co‐occurrences through probability theory, following Bowers and Brown (1982) and Veech (2006), and then calculated the species associations (RII) using the formula described by Armas, Ordiales, and Pugnaire (2004) (Formula S1).

To select the significant pairs, we developed a pairwise implementation of null models using the “curveball algorithm” or sim9 in the R package *EcoSimR* (Gotelli, Hart, and Ellison 2015; Strona et al. 2014). We used 1000 randomizations and 5 N for the number of swaps, following the rule of thumb of > 4 N (more than four times the total number of links in the network; Maslov and Sneppen 2002; Neal et al. 2023). *p‐*values were obtained by calculating the upper and lower tail probabilities by taking the average number of times that the RII of the observed matrix was higher or lower than the RII in 1000 random matrices. For the probabilistic method, we used effect sizes standardized by the number of sites as a measure of co‐occurrence intensity. The probability of co‐occurrence at a frequency greater than the observed frequency was used as a measure of significance and calculated analytically using the hypergeometric distribution (Formula S2). In addition to the RII method, we chose the probabilistic method because it was found to be more effective than other pairwise co‐occurrence tests in a recent study that used simulated data (Lavender et al. 2019).

We calculated pairwise scores for plant and butterfly abundance data using Spearman correlation, a rank‐based non‐parametric test, that relaxes the normality assumption made by Pearson correlation at the cost of losing information, but that has been shown to perform slightly better than Pearson correlation for mutualistic interactions (Weiss et al. 2016). To reduce the false discovery rate, *p*‐values were adjusted for multiple testing using the Benjamini–Hochberg method (Benjamini and Hochberg 1995).

### 2.4 Accuracy Analysis

**FIGURE 1:**
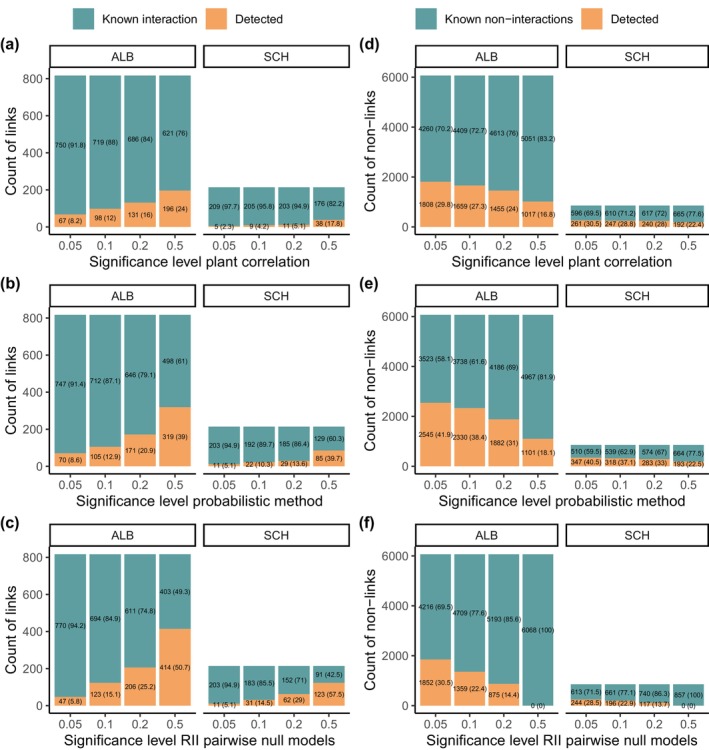
Sensitivity (a–c) and specificity (d–f) per region at different significance values for the three tested co‐occurrence methods: Plant correlation, Probabilistic, and RII null models. Sensitivity (a–c) was transformed to a percentage and appears in parentheses within the “detected” orange bar (blue/green bars represent the undetected known interactions). It represents the probability of detecting a true link and is calculated as TP/(TP + FN). The known links are the 1 s (links) in the interaction matrix. Specificity (d–f) was transformed into a percentage and appears in parentheses within the “detected” orange bar (blue/green bars represent the undetected known non‐interactions). Specificity was defined as the true negative rate and was calculated as TN/(TN + FP). Known non‐links are the 0 s in the interaction matrix. Abbreviations: ALB = region Swabian Jura; FN = false negatives; FP = false positives; Plant correlation = Spearman's rank correlation with Benjamini–Hochberg's multiple testing correction; Probabilistic = probabilistic method (Veech 2013); RII null models = Relative Interaction Intensity index with pairwise null models (Armas, Ordiales, and Pugnaire 2004); SCH = region Schorfheide‐Chorin; TN = true negatives; TP = true positives.

To compare the different methods, we calculated the sensitivity and specificity ratios of the co‐occurrence algorithm results obtained at different significance cutoff values (0.05, 0.1, 0.2, and 0.5). For this, we first classified pairs of plants and butterflies as true positives (TP), that is, the number of ecological interactions that showed a positive spatial association; true negatives (TN), the number of non‐interactions that did not show a significant spatial association; false positives (FP), the number of interactions that did not show a significant spatial association; and false negatives (FN), the number of non‐interactions that showed a significant spatial association (Figures S3 and S4). Then, we calculated the sensitivity, which is defined as the probability of detecting a true link and is calculated as TP/(TP + FN), whereas specificity was defined as the TN rate and was calculated as TN/(TN + FP). Both sensitivity and specificity ranged from 0 to 1, with higher values indicating a higher likelihood of discriminating between true and false interactions. The significance level for the sensitivity and specificity is represented by ⍺ and β, respectively. To compare the methods, we chose a significance level of 0.2, because it was a good compromise between high sensitivity and acceptable specificity (Figure 1) and because our main goal was to maximize the detected interactions. At this significance level, the final numbers of TP and TN achieved with each method were compared using Pearson's chi‐squared test of independence (*X* ^2^). This test assumes that cases are randomly sampled from the population and that non‐occurrences are included; therefore, we also used FN in the test. Due to the nature of mutualistic interactions, only significant positive associations were considered, and negative associations were considered zero. Additionally, we calculated the number of potential interactions (total number of species on the lower taxa times the total number of species on the higher taxa), realized interactions (number of TP at the selected ⍺ level), and connectance (relative number of interactions over the maximum number of potential interactions) for each of the methods. All scripts for analyses can be found in the repository https://doi.org/10.5281/zenodo.10931678.

### 2.5 Flower Availability Data and Data Validation

**FIGURE 2:**
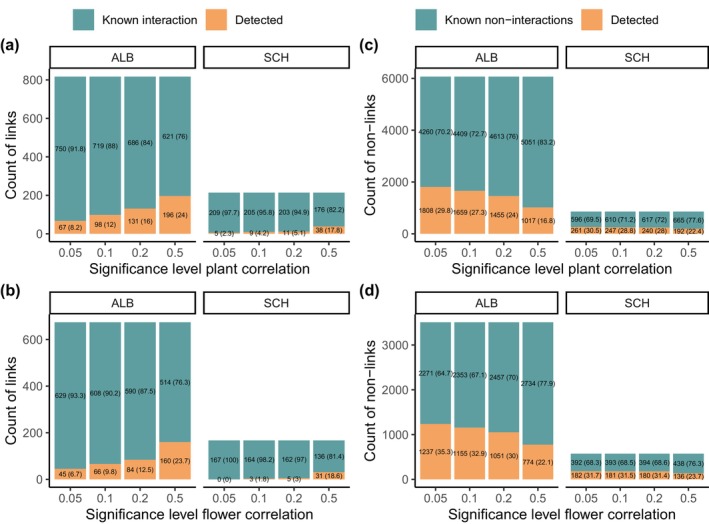
Sensitivity (a, b) and specificity (c, d) per region at different significance values of the Spearman's rank correlation method for the abundance of lepidopteran species with the abundance of plants (a and c) and flowers (b and d) at different significance levels. Sensitivity was transformed to a percentage and appears in parentheses within the “detected” orange bar (blue/green bars represent the undetected known interactions). Sensitivity represents the probability of detecting a true link and was calculated as TP/(TP + FN). Known links are any values > 0 in the interaction matrix. Specificity was transformed to percentage and appears in parentheses within the “detected” orange bar (blue/green bars represent the undetected known non‐interactions). It was defined as the true negative rate and was calculated as TN/(TN + FP). Known non‐links are the 0 s in the interactions matrix. Abbreviations: ALB = region Swabian Jura; FN = false negatives; FP = false positives; Plant, and Flower correlation = Spearman's rank correlation of plants and flowers with Benjamini–Hochberg's multiple testing correction; Probabilistic = probabilistic method (Veech 2013); RII null models = Relative Interaction Intensity index with pairwise null models (Armas, Ordiales, and Pugnaire 2004); SCH = region Schorfheide‐Chorin; TN = true negatives; TP = true positives.

To control for possible phenological mismatches between plants and Lepidoptera and ensure that flowers and pollinators were active at the same time, we calculated co‐occurrences with Spearman's rank correlation method using the abundance of Lepidoptera with the “abundance” of flowers (i.e., flower availability), instead of just vegetation abundance, therefore allowing only for interactions between flowers and pollinators (instead of plants and pollinators) and compared them (Figure 2). We used flower availability datasets from BExIS (datasets 4981 and 4964; Weiner, Linsenmair, and Blüthgen 2019a, 2019b). Flower data were gathered between April and September 2008 by recording all flowering plant species and by counting or estimating the number of flowering units per species. A flower unit was defined as one or more flowers that demanded insects to fly from one unit to another. To prepare the data, we calculated the average flowering units for each species and site across all dates. We then checked and removed the NAs (i.e. sites where either plants or butterflies were not sampled) and subsets of plants that did not occur in the occurrence and ecological interaction datasets. We also log(*x* + 1)‐transformed the flower numbers to avoid a high range of values. We used the correlation coefficients to select the positive correlations and corrected the *p*‐values using the Benjamini–Hochberg method to retain only significant associations.

In addition to the occurrence data obtained from empirical surveys and ecological interaction data obtained from the literature, we extracted flower visitation data from BExIS to validate our results. These data were recorded between April and August 2008 (BExIS dataset 10,160; Weiner et al. 2016). Flower visits were recorded over 6 h on a 600 m^2^ transect (subdivided into smaller intercepts) around each grassland plot. Only insects sitting directly in the center of flowers that appeared to feed on pollen or nectar were caught. Insects resting on petals were not sufficient for inclusion. We removed family‐ and genus‐level observations and calculated the total sum of butterfly flower visits per plant species and region across all sites, dates, and intercepts. Using only the pairs of plants and butterflies for which we had flower visitation data, we checked the following: (1) the number and percentage of negative co‐occurrences found by any of the association methods (Table S1), (2) the correlation between the number of flower visits and the positive association values obtained with each co‐occurrence method using simple linear models for each method (Tables S2 and S3; Figure S5), and (3) the correlation between flower visits and species interactions of plants and butterflies using Spearman's rank correlation (Table S4). For 1 and 2, we used a significance value of 0.2 to consider co‐occurrence as significant because we wanted to find as many pairs of species as possible and because this was the cutoff found to be sufficient in our accuracy analysis.

### 2.6 Interaction Strength Signal

**FIGURE 3:**
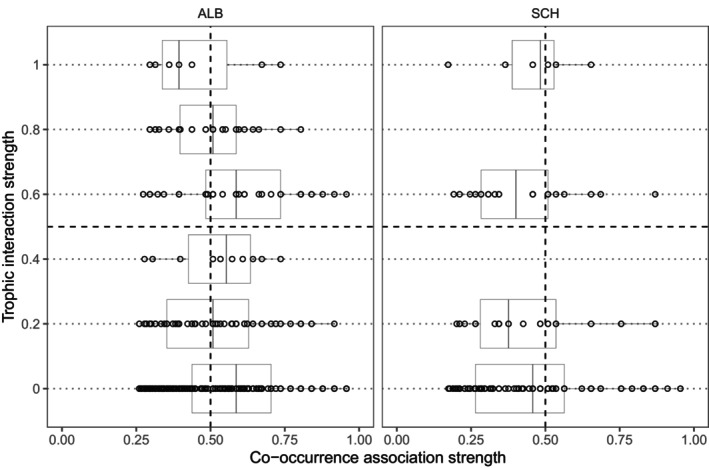
Quadrant analysis showed incongruence between the actual interaction strength and predicted co‐occurrence association strength per region. Each empty point represents a pair of plants and butterfly species. To aid visualization, we added dashed black lines that divide each plot into four quadrants and thin dotted black lines for each trophic level. At specified interaction strengths of 0 (low), 0.2, 0.4, 0.6, 0.8, and 1 (strong), the boxes show the interquartile range, that is, the first and third quartiles (the 25th and 75th percentiles).

To explore how the strength of the interaction signal is translated into co‐occurrence data, we represented it in a coordinate system by plotting the intensity of co‐occurrence on the *x*‐axis, which is the strength of all plant‐butterfly pair associations, against the strength of species interaction on the *y*‐axis, and dividing the space into four quadrants (Figure 3). The lower left quadrant represents pairs with weak (or no) trophic interactions and weak (or no) co‐occurrence, which might arise from the interactions themselves; for example, butterflies might avoid unpalatable plants. On the upper right quadrant, pairs with strong trophic interaction and strong co‐occurrence indicate strong, positive interactions, which are independent of other filters (Ovaskainen, Hottola, and Siitonen 2010). On the lower right quadrant, pairs with weak (or no) trophic interaction but strong co‐occurrence (and therefore strong spatial aggregation) might result from shared preferences for environmental factors or environmental filtering (Peres‐Neto, Olden, and Jackson 2001) or simply because these species have higher local intrinsic growth rates (Cadotte and Tucker 2017) and dispersal limitation (Ulrich 2004), although this might not apply for all butterfly species, or spatial variability in dispersal and subsequent settlement and recruitment (de Bello et al. 2012). Finally, in the upper‐left quadrant, pairs with strong trophic interactions but low (or no) co‐occurrence might simply indicate that spatial association is a poor proxy for ecological interactions (Blanchet, Cazelles, and Gravel 2020). In addition to plotting the strength of co‐occurrence association and the strength of interaction, we ran an Ordinal Logistic Regression (OLR) model using the MASS package in R (Venables and Ripley 2002) using only pairs of plants and butterflies with positive co‐occurrences (using the RII index with pairwise null models). Only the model for region ALB was significant, where the probability of a pair of interacting species having a high interaction strength decreased with a higher co‐occurrence association, especially at higher levels of interaction strength, but the model did not meet with the proportional odds assumption; therefore, this model should be interpreted with caution and should only be considered as a preliminary insight into the relationship between co‐occurrence association and interaction strength (Figure S6; Tables S5 and S6). Additionally, to further validate these results and because we only had one predictor, we conducted a one‐way ANOVA to compare the differences in means of co‐occurrence association strength (continuous variable) for each interaction strength (categorical variable). We further used a Tukey HSD post hoc test with a 95% CI to compare all possible group pairings (Figure S7).

## 3 Results

For the ALB and SCH regions, 51 and 21 lepidopteran and 135 and 51 plant species, respectively (Figures S1 and S2), that simultaneously occurred in the field observations (abundance data) and showed a mutualistic interaction in the literature (species interactions) were selected.

The comparison of pairwise associations obtained from co‐occurrence analyses with species interaction data showed that, particularly at lower ⍺ levels, where the models had lower levels of “false positives” (Table 1), all tested methods had low sensitivity (Figure 1a–c). The best performing method was the implementation of the RII index with pairwise null models. The second‐best performing method was the probabilistic model, and the worst performing method was Spearman's rank correlation method with multiple testing correction. At a significance level of *⍺* = 0.2, the difference in the number of correctly detected interactions between the two methods was significant for ALB (*X* ^2^ = 3.986, df = 1, *N* = 817, *p* = 0.046) and SCH (*X* ^2^ = 14.291, df = 1, *N* = 214, *p* < 0.001). At this significance level, the sensitivity values achieved were 0.252, 0.209, and 0.160 using methods RII with pairwise null models, Probabilistic, and Spearman's rank correlation, respectively.

**TABLE 1:** Details of the sensitivity and specificity analyses for all co‐occurrence methods at each significance level.

Method	Region	*⍺*	TP	TN	FP	FN	Sensitivity	Specificity
RII	ALB	0.050	47	1852	4216	770	0.058	0.305
RII	ALB	0.100	123	1359	4709	694	0.151	0.224
RII	ALB	0.200	206	875	5193	611	0.252	0.144
RII	ALB	0.500	414	0	6068	403	0.507	0.000
RII	SCH	0.050	11	244	613	203	0.051	0.285
RII	SCH	0.100	31	196	661	183	0.145	0.229
RII	SCH	0.200	62	117	740	152	0.290	0.137
RII	SCH	0.500	123	0	857	91	0.575	0.000
Probabilistic	ALB	0.050	70	2545	3523	747	0.086	0.419
Probabilistic	ALB	0.100	105	2330	3738	712	0.129	0.384
Probabilistic	ALB	0.200	171	1882	4186	646	0.209	0.310
Probabilistic	ALB	0.500	319	1101	4967	498	0.390	0.181
Probabilistic	SCH	0.050	11	347	510	203	0.051	0.405
Probabilistic	SCH	0.100	22	318	539	192	0.103	0.371
Probabilistic	SCH	0.200	29	283	574	185	0.136	0.330
Probabilistic	SCH	0.500	85	193	664	129	0.397	0.225
Plant cor	ALB	0.050	67	1808	4260	750	0.082	0.298
Plant cor	ALB	0.100	98	1659	4409	719	0.120	0.273
Plant cor	ALB	0.200	131	1455	4613	686	0.160	0.240
Plant cor	ALB	0.500	196	1017	5051	621	0.240	0.168
Plant cor	SCH	0.050	5	261	596	209	0.023	0.305
Plant cor	SCH	0.100	9	247	610	205	0.042	0.288
Plant cor	SCH	0.200	11	240	617	203	0.051	0.280
Plant cor	SCH	0.500	38	192	665	176	0.178	0.224
Flower cor	ALB	0.050	45	1237	2271	629	0.067	0.353
Flower cor	ALB	0.100	66	1155	2353	608	0.098	0.329
Flower cor	ALB	0.200	84	1051	2457	590	0.125	0.300
Flower cor	ALB	0.500	160	774	2734	514	0.237	0.221
Flower cor	SCH	0.050	0	182	392	167	0.000	0.317
Flower cor	SCH	0.100	3	181	393	164	0.018	0.315
Flower cor	SCH	0.200	5	180	394	162	0.030	0.314
Flower cor	SCH	0.500	31	136	438	136	0.186	0.237

Abbreviations: *⍺* = significance level, ALB = Region Swabian Jura, FN = false negatives, FP = false positives, Plant and Flower cor = Spearman's rank correlation of plants and flowers with Benjamini–Hochberg's multiple testing correction, Probabilistic = probabilistic method (Veech 2013), RII = Relative Interaction Intensity index with pairwise null models (Armas, Ordiales, and Pugnaire 2004), SCH = region Schorfheide‐Chorin, TN = true negatives, TP = true positives.

The highest specificity was achieved using the probabilistic method of Veech (2013, 2014). The RII with pairwise null models and the Spearman's rank correlation methods had similar specificity at *β* = 0.05, but the specificity of the RII decreased strongly with the increasing significance level (Figure 1e,f). In comparison, the specificity of the Spearman's rank correlation method was more constant and decreased less strongly (Figure 1d); thus, at the *β* = 0.2 level, the specificity of the Spearman method was 10% higher than that of RII. At this selected level of significance, the number of TN detected with the probabilistic method was significantly higher than the RII method in ALB (*X* ^2^ = 474.98, df = 1, *N* = 817, *p* = 0.001) and SCH (*X* ^2^ = 88.782, df = 1, *N* = 214, *p* < 0.001).

At the selected significance level of *⍺* = 0.2, the number of realized and relative number of interactions achieved by any of the methods was significantly lower than those of the empirical data in both regions (Table 2). In particular, the connectance achieved with the best performing method (RII with pairwise null models) was 4.0 and 3.5 times less than the empirical connectance in ALB and SCH respectively.

**TABLE 2:** Network‐level summary metrics for the observed community of species interactions and for the reconstructed associations using co‐occurrence analyses with a significance level of *⍺* = 0.2.

Method	Region	Potential associations	Realized associations	Connectance
**Empirical**	**ALB**	**6885**	**817**	**0.119**
RII null models	ALB	6885	206	0.030
Probabilistic	ALB	6885	171	0.025
Plant correlation	ALB	6885	131	0.019
Flower correlation	ALB	4182	84	0.020
**Empirical**	**SCH**	**1071**	**214**	**0.200**
RII null models	SCH	1071	62	0.058
Probabilistic	SCH	1071	29	0.027
Plant correlation	SCH	1071	11	0.010
Flower correlation	SCH	741	5	0.007

*Note:* Values in bold highlight those in the (known) empirical community.

Abbreviations: ALB = Swabian Jura, Plant and Flower correlation = Spearman's rank correlation of plants and flowers with Benjamini–Hochberg's multiple testing correction, Probabilistic = probabilistic method (Veech 2013), SCH = Schorfheide‐Chorin, RII null models = Relative Interaction Intensity index with pairwise null models (Armas, Ordiales, and Pugnaire 2004).

Running the co‐occurrence analyses with the abundance of flowers instead of just plant abundance did not improve the sensitivity (Figure 2a,b). At the *⍺* = 0.2 level, the number of TP identified for plants and flowers did not differ significantly neither in ALB (*X* ^2^ = 3.533, df = 1, *N* = 817, *p* = 0.060) nor in SCH (*X* ^2^ = 0.607, df = 1, *N* = 214, *p* < 0.436). In contrast, specificity was improved (Figure 2c,d) for the ALB region (*X* ^2^ = 40.859, df = 1, *N* = 6885, *p* < 0.001), but not for the SCH region (*X* ^2^ = 1.707, df = 1, *N* = 1071, *p* = 0.191).

The analysis of quadrants for species pairs using the relationship between interaction strength and co‐occurrence association strength revealed a high degree of incongruence between the predicted association strength of the best performing co‐occurrence method and the actual species interaction strength in both regions (Figure 3). Specifically, a high number of pairs had high interaction strength but low co‐occurrence association and vice versa. The one‐way ANOVA showed that there was a statistically significant difference in co‐occurrence association strength between at least two of the levels of interaction strength only for region ALB (*F* (5) = 4.098, *p* = 0.001), but not for region SCH (*F* (3) = 0.180, *p* = 0.910). The Tukey HSD post hoc test revealed that this difference was between species interaction strength levels 0 and 0.2 (Figure S7).

## 4 Discussion

In this study, we investigated the performance of three pairwise co‐occurrence methods for detecting mutualistic species interactions between butterflies and flowering plant species in temperate grassland ecosystems. Given the results of prior investigations, which revealed that co‐occurrence methods are more effective in detecting positive interactions than negative ones, we anticipated that the presence of associations would be evident in the P/A and abundance data, and that these signals would become more pronounced when using additional flower abundance data.

Contrary to our expectations, all three methods showed weak performances. Among them, the RII index with pairwise null models performed best for sensitivity, whereas the probabilistic method showed the highest specificity. However, all the methods exhibited low sensitivity, particularly at higher significance levels, affecting their ability to accurately detect interactions. In a study on marine intertidal ecosystems by Freilich et al. (2018), at an *⍺* = 0.1, the sensitivity (0.692) was higher than that of ALB (0.129) and SCH (0.103) using the probabilistic model (sensu Veech 2013), but the specificity (0.283) was lower than that of ALB (0.384) and SCH (0.371). This could be explained by: (1) the sessile nature of marine intertidal species compared with the relatively high mobility of butterflies (Gullan and Cranston 2014), for which interactions between sessile organisms might leave a stronger signal in the occurrence and abundance data; (2) butterflies might not be efficient pollinators (Jennersten 1984), even though it has been shown that some butterflies pollinate some herbaceous plants in temperate climates, such as northern Europe (Table 1 in Andersson et al. 2002), and are specially adapted for long‐distance pollination (Courtney, Hill, and Westerman 1982); (3) some butterfly species may exhibit limited flower constancy, which can fluctuate based on local flower availability (Szigeti et al. 2018) and even change during the lifetime of individual butterflies (Szigeti et al. 2019); and (4) possibly, the relatively limited sampling effort for the occurrence data (approximately one sample per month per site). Following the logic of points one and three and given that butterfly larvae show more specific interactions with their larval host plants and are less mobile than adults (Ebert and Rennwald 1991), one would expect stronger patterns of species co‐occurrence between them, resulting in higher sensitivity and specificity.

At a significance level of *⍺* = 0.2, the best method performed significantly better than the second‐best method. Although the chosen significance level (*⍺* = 0.2) aimed for the highest sensitivity and acceptable specificity, the sensitivity values were modest at the conventional *⍺* = 0.05. Despite achieving higher specificity, none of the methods detected as many interactions as the empirical data at any of the tested significance levels (range from 0.05–0.5). Detection was not low because there were extremely few or high numbers of true interactions in the dataset. In this respect, the relative number of interactions of the empirical datasets extracted from the literature for region ALB (0.119) and SCH (0.2) were within the values of other empirical datasets typically found in the literature which range (on average) between 0.1 and 0.3 (Dunne, Williams, and Martinez 2002; Rezende, Jordano, and Bascompte 2007; Baumgartner 2020). This supports the validity of using interaction data from the literature in this study.

Including the abundance of flowers in the analyses, rather than the abundance of plants, did not improve sensitivity (Figure 2a,b) but did improve specificity within the ALB region (Figure 2c,d). These findings underscore the complexity of ecological interactions and emphasize the importance of considering regional differences and environmental contexts when interpreting the results. Further research is needed to refine the methodologies for the accurate assessment of species interactions in diverse ecological settings.

Our study also aimed to explore the relationship between the signal strength of species interactions and the co‐occurrence patterns in ecological systems. Through various analytical methods, including plotting, OLR, and one‐way ANOVA, we sought to understand how the strength of interactions influences co‐occurrence associations. However, our results revealed significant discrepancies between the predicted and observed patterns in both regions, particularly in the disproportionate number of species pairs exhibiting high interaction strength but low co‐occurrence, and vice versa. Moreover, the negative relationship found in ALB (whereas no relationship was found in SCH) suggests the influence of local environmental factors and regional management on the observed patterns. In the SCH region, historically intense local management has reduced local species richness and simplified the regional community to mostly generalist species in comparison to region ALB (Gelbrecht et al. 2016). Based on the Species Interactions–Abiotic Stress Hypothesis (SIASH), the effect of species interactions on occurrence (and therefore the extent of their geographical ranges) would also differ across abiotic stress gradients (Louthan, Doak, and Angert 2015), with the intensity or number of interactions per interactor being higher in high‐stress environments. These findings highlight the complexity of ecological dynamics and emphasize the limitations of using co‐occurrence as a proxy for species interactions.

In our analysis, the implementation of the RII metric (Armas, Ordiales, and Pugnaire 2004) with the pairwise null model using the “curveball algorithm” (Strona et al. 2014) was the method with the highest sensitivity. This algorithm works by fixing the row and column totals, that is, perfectly constraining the matrix, with the marginal totals of the null matrix matching those of the original matrix (Strona et al. 2014). Fixing marginal totals not only has been shown to reduce the risk of type II statistical error (Ulrich and Gotelli 2013), but also has ecological implications. For example, in a species‐by‐site matrix (rows × columns), fixing the row totals means that we maintain species occurrence across sites; hence, species that occupy many or few sites represent generalists or specialists. Conversely, when fixing column totals, we maintain the occupancy of sites; hence, sites with high occupancy also represent sites with high resource availability (Gotelli and Graves 1996). In the context of our analysis, this would also maximize the chances of preserving species interactions across sites, helping to increase their detection. Freilich et al. (2018) found that, especially for positive interactions, sensitivity increases rapidly when species become more common; hence, implementing a model that allows for representation of this might also increase the chances of detection.

In contrast, the probabilistic model was the second best in detecting true interactions (sensitivity) but the best in detecting the absence of interactions (highest specificity). Arita (2016) pointed out that the probabilistic method corresponds to Fisher's exact test, therefore being the analytical equivalent to a null model with fixed marginal rows but unconstrained columns (i.e., a F‐E null model). This means that the probability of a cell in the null matrix is only dependent on the corresponding row total, but independent of the column totals of the original matrix (Veech 2013); hence, all sites have the same probability of being occupied by a given species. Butterfly species have a high dispersal ability (Ebert and Rennwald 1991). Therefore, they could fly and possibly occupy many sites; therefore, representing this trait using this null model could help increase the sensitivity and especially the specificity of the model.

Nevertheless, with any of the methods used, the specificity was generally higher than the sensitivity, showing the superior capabilities of the methods for detecting true absences of interactions over the existence of true interactions. This suggests that, while these methods provide insights, they do not capture the complexity of real ecological interactions, especially when these mechanisms do not leave such a strong spatial signal. In recent years, the analysis of co‐occurrences and interpretation of significant associations has also been criticized (Blanchet, Cazelles, and Gravel 2020; Goberna and Verdú 2022). Caution should be taken when directly interpreting significant associations (aggregation or segregation) as ecological interactions because other factors could influence the detected pattern. For example, co‐occurrence patterns might arise due to dispersal limitations or environmental factors hindering the distinction between the influence of species interactions and the environment based on species distribution data (Gotelli and Ulrich 2012; Godsoe, Franklin, and Blanchet 2016). Furthermore, for species with shared phenotypic attributes and small niche differences, these species may co‐occur simply because they have higher local intrinsic growth rates than other species (Cadotte and Tucker 2017). Interactions with other species, indirect species interactions, and increased matrix complexity may also hamper detection (Cazelles et al. 2016). Another consideration is that to correctly interpret ecological interactions, the spatial scale of the analysis should always match the scale of the interactions and the analysis should consider a large sample size (Blanchet, Cazelles, and Gravel 2020). Moreover, to detect patterns, biotic interactions should first leave a signal in the data, which is usually assumed in analyses and might be less pronounced in highly mobile organisms.

Our study showed the shortcomings of prevalent co‐occurrence methods in detecting mutualistic ecological interactions for highly mobile species, underscoring the need for refined methodologies or abandoning such long‐sought shortcuts. We emphasize the importance of understanding algorithmic implications, caution against direct interpretation of co‐occurrence patterns as interactions, and advocate refined approaches and consideration of contextual factors to better comprehend ecological interactions.

## Author Contributions

**Esteban Menares:** data curation (equal), formal analysis (lead), investigation (equal), methodology (equal), software (lead), visualization (lead), writing – original draft (lead), writing – review and editing (equal). **Hugo Saíz:** formal analysis (supporting), methodology (equal), software (supporting), supervision (equal), writing – review and editing (equal). **Noëlle Schenk:** formal analysis (supporting), software (supporting), writing – review and editing (equal). **Enrique G. de la Riva:** supervision (equal), writing – review and editing (equal). **Jochen Krauss:** data curation (equal), investigation (equal), writing – review and editing (equal). **Klaus Birkhofer:** conceptualization (lead), funding acquisition (lead), methodology (equal), project administration (lead), supervision (equal), writing – review and editing (equal).

## Ethics Statement

Fieldwork permits for the original data collection obtained from BExIS were issued by the responsible state environmental offices of Baden‐Württemberg, Thüringen, and Brandenburg. We thank the administration of the Hainich national park, the UNESCO Biosphere Reserve Swabian Alb and the UNESCO Biosphere Reserve Schorfheide‐Chorin as well as all landowners for the excellent collaboration.

## Conflicts of Interest

The authors declare no conflicts of interest.

## Supporting information


Data S1.

## Data Availability

The datasets generated in this study are publicly available on Biodiversity Exploratories Information System (BExIS; http://doi.org/10.17616/R32P9Q) under IDs 31,733, 31,734, 31,735, 31,736, 31,737, and 31,738. This study was based on data from several projects in the Biodiversity Exploratories program (DFG Priority Program 1374) (Weiner, Linsenmair, and Blüthgen 2019a, 2019b; Schäfer 2018; Börschig and Krauss 2016; Weiner et al. 2016). All scripts for analyses can be found in the repository https://doi.org/10.5281/zenodo.10931678.
