# Supplementary material for: Co‐Occurrence Patterns Do Not Predict Mutualistic Interactions Between Plant and Butterfly Species
Source: Ecol Evol. 2024 Oct 30;14(11):e70498. doi: 10.1002/ece3.70498 (PMC11525043; doi:10.1002/ece3.70498)
Supplement: Supplementary file 1 — Data S1. [file ECE3-14-e70498-s001.docx]

**Supplemental Information for:**

**Co-occurrence patterns do not predict mutualistic interactions between plant and butterfly species.**

Esteban Menares, Hugo Saíz, Noëlle Schenk, Enrique G. de la Riva, Jochen Krauss, Klaus Birkhofer

**Table of Contents:**

| **Appendix S1:** Heatmaps of species interactions and accuracy of co-occurrence prediction per species | Page 2 |
| --- | --- |
| **Appendix S2:** Mathematical formulas to calculate species associations for RII index (Armas et al. 2004) and Probabilistic Method (Veech, 2013 and 2014) | Page 4 |
| **Appendix S3:** Data validation using flower visitation data | Page 5 |
| **Appendix S4:** interaction strength test model summaries and ANOVA post-hoc test | Page 8 |
| **References** | Page 10 |

**Appendix S1:** Heatmaps of species interactions.

**Figure S1.** Heatmap of (vascular) plant and butterfly species interactions in grasslands at region ALB.

**Figure S2.** Heatmap of (vascular) plant and butterfly species interactions in grasslands at region SCH.

**Figure S3.** Heatmap per species of the accuracy of co-occurrence predictions using the Relative Interaction Intensity index with pairwise null models with a significance level ≤ 0.2 in region ALB.

**Figure S4.** Heatmap per species of the accuracy of co-occurrence predictions using the Relative Interaction Intensity index with pairwise null models with a significance level ≤ 0.2 in region SCH.

**Appendix S2:** Mathematical formulas to calculate species associations for RII index (Armas et al. 2004) and Probabilistic Method (Veech, 2013 and 2014)

**Formula S1:** RII.

$$RII=\frac{B_{w}- B_{o}}{B_{w}+B_{o}}= \frac{Observed- Expected}{Observed+Expected}$$

**Formula S2:** Probabilistic method.

$$p_{j}= \frac{\left( \begin{matrix} N \\ j \end{matrix} \right) \times\left( \begin{matrix} N - j \\ N_{2} - j \end{matrix} \right) \times\left( \begin{matrix} N - N_{2} \\ N_{1} - j \end{matrix} \right)}{\left( \begin{matrix} N \\ N_{2} \end{matrix} \right) \times\left( \begin{matrix} N \\ N_{1} \end{matrix} \right)}$$

Where:

$p_{j}$ : probability that species 1 and 2 co-occur at exactly j sites

$\left( \begin{matrix} N \\ j \end{matrix} \right)$: # of ways j sites can be arranged among N sites

$\left( \begin{matrix} N - j \\ N_{2} - j \end{matrix} \right)$ : # of ways species 2 can be arranged in sites that don’t have both species

$\left( \begin{matrix} N - N_{2} \\ N_{1} - j \end{matrix} \right)$ : # of ways species 1 can be arranged among sites that don’t have species 2

$\left( \begin{matrix} N \\ N_{2} \end{matrix} \right)$ : # of ways species 2 can be arranged

$\left( \begin{matrix} N \\ N_{1} \end{matrix} \right)$ : # of ways species 1 can be arranged

**Appendix S3:** Data validation using flower visitation data.

**Table S1.** The percentage (and number) of negative co-occurrences identified by each association method per region, only for pairs with flower-visiting data used for data validation.

| Region | Count of pairs | RII pairwise null models | Probabilistic | Plant correlation | Flower correlation |
| --- | --- | --- | --- | --- | --- |
| ALB (southwest) | 85 | 0 | 5.9% (n = 5) | 2.4% (n = 2) | 0 |
| SCH (northeast) | 18 | 0 | 5.6% (n = 1) | 0 | 0 |

**Notes**: ALB = region Schwäbische Alb; SCH = region Schorfheide-Chorin; RII = Relative Interaction Intensity index with pairwise null models (Armas, Ordiales and Pugnaire, 2004); Probabilistic = probabilistic method (Veech, 2013); Plant and Flower cor = Spearman’s rank correlation of plants and flowers with Benjamini-Hochberg’s multiple testing correction. Negative co-occurrences were identified using the sign of the association in conjunction with significance values. For RII, p-values were obtained by calculating the upper and lower tail probabilities by taking the average of times the RII of the observed matrix was higher or lower than the RII in the 1000 random matrices. For the probabilistic method, we used the P_lt_ and P_gt_ values obtained using the hypergeometric distribution with the *cooccur* package (Griffith, Veech and Marsh, 2016). For plant and flower correlations, we used the p-value corrected for multiple testing using the Benjamini and Hochberg’s correction. Significance level was set to ⍺ = 0.2 in all cases.

**Table S2.** Summary of linear models between flower visits and the (significant) positive association values obtained with the different co-occurrence methods of pairs of plants and butterflies in region ALB. The lower and upper confidence interval of the estimate is shown in parenthesis.

|  | **Plant cor - ALB** | | **Flower cor - ALB** | | **Probabilistic - ALB** | | **RII - ALB** | |
| --- | --- | --- | --- | --- | --- | --- | --- | --- |
| *Predictors* | *Estimates* | *p* | *Estimates* | *p* | *Estimates* | *p* | *Estimates* | *p* |
| Intercept | 0.42 (0.36 – 0.49) | **<0.001** | 0.46 (0.40 – 0.52) | **<0.001** | 0.05 (0.04 – 0.06) | **<0.001** | 0.51 (0.43 – 0.59) | **<0.001** |
| Flower visits | 0.01 (0.00 – 0.03) | 0.088 | 0.02 (0.00 – 0.03) | **0.037** | 0.00 (0.00 – 0.01) | **0.044** | 0.01 (-0.02 – 0.03) | 0.500 |
| Observations | 29 | | 19 | | 36 | | 28 | |
| R^2^ | 0.104 | | 0.231 | | 0.114 | | 0.018 | |

**Table S3.** Summary of linear models between flower visits and the (significant) positive association values obtained with the different co-occurrence methods of pairs of plants and butterflies in region SCH. The lower and upper confidence interval of the estimate is shown in parenthesis.

|  | **Probabilistic - SCH** | | **RII - SCH** | |
| --- | --- | --- | --- | --- |
| *Predictors* | *Estimates* | *p* | *Estimates* | *p* |
| Intercept | 0.07 (0.03 – 0.10) | 0.006 | 0.28 (0.14 – 0.41) | 0.005 |
| Flower visits | 0.00 (-0.01 – 0.01) | 0.917 | -0.01 (-0.05 – 0.03) | 0.530 |
| Observations | 6 | | 6 | |
| R^2^ | 0.003 | | 0.106 | |

**Figure S5.** Scatterplot between flower visits and (significant) positive associations obtained with the three methods: RII with pairwise null models, Veech’s probabilistic, and Spearman’s rank correlation with Benjamini and Hochberg’s correction for multiple testing. The line is a best fit line from a linear model.

**Table S4.** Spearman’s rank correlation of flower visits and ecological interaction intensity of pairs of plant and butterfly for which both kinds of data were available per region. One-sided test (alternative = greater), for positive associations.

| Region | Estimate | Statistic | p-value | Method | Alternative |
| --- | --- | --- | --- | --- | --- |
| ALB | 0.364 | 65098.813 | < 0.001 | Spearman's rank correlation rho | greater |
| SCH | -0.058 | 1025.244 | 0.59 | Spearman's rank correlation rho | greater |

**Appendix S4:** Interaction strength Ordinal Logistic Regression (OLR) and (inverted) ANOVA model summaries and ANOVA post-hoc.

We ran one ORL model per region, using co-occurrence association strength (continuous variable) as a predictor and interaction strength (categorical variable) as a response. To test the significance of the models, we calculated p-values by comparing the t-value against the standard normal distribution and calculating the confidence intervals (CI) for the parameter estimates. These can be obtained using the standard errors and assuming a normal distribution, where if the 95% CI does not cross 0, the parameter estimate is statistically significant. Only the model for region ALB was significant, where the probability of a pair of interacting species having a high interaction strength decreases with a higher co-occurrence association, especially at higher levels of interaction strength, but the model did not comply with the proportional odds assumption; therefore, this model should be interpreted with caution and regarded only as a preliminary insight into the relationship of co-occurrence association and interactions strength. As the estimates in the output of the model are given in units of ordered logits, which are challenging to interpret because they are scaled in terms of logs, we converted the coefficients and confidence intervals into odds ratios by exponentiating them. The OLR model suggest that the probability of a pair of interacting species with high interaction strength decreased with higher co-occurrence association, especially at higher levels of interaction strength within the ALB region. For every one-unit increase in the co-occurrence value of a pair (i.e., the association strength), the odds of having a higher interaction strength are multiplied by 0.245 (95% CI [0.102, 0.582]); that is, decreases by 75.5%.

**Figure S6.** The probability of a pair of interacting species having a high interaction strength decreases with a higher co-occurrence association, especially at higher levels of interaction strength. Ordinal Logistic Regression (OLR) model for region ALB using co-occurrence association strength (continuous variable) as a predictor and interaction strength (categorical variable) as a response. Co-occurrence association strengths were obtained using the RII index with pairwise null models using a significance cut-off of 0.2.

**Table S5.** OLR model summaries for interaction strength test.

OLR - ALB

|  | Value | Std. Error | t value | p-value |
| --- | --- | --- | --- | --- |
| Co-occurrence association strength | -1.409 | 0.445 | -3.166 | 0.002 |
| 0\|0.2 | 1.172 | 0.248 | 4.72 | <0.001 |
| 0.2\|0.4 | 2.035 | 0.259 | 7.867 | <0.001 |
| 0.4\|0.6 | 2.154 | 0.261 | 8.249 | <0.001 |
| 0.6\|0.8 | 3.061 | 0.291 | 10.516 | <0.001 |
| 0.8\|1 | 4.689 | 0.446 | 10.519 | <0.001 |

OLR – SCH

|  | Value | Std. Error | t value | p-value |
| --- | --- | --- | --- | --- |
| Co-occurrence association strength | -0.427 | 0.742 | -0.576 | 0.565 |
| 0\|0.2 | 0.8 | 0.361 | 2.215 | 0.027 |
| 0.2\|0.6 | 1.627 | 0.378 | 4.3 | <0.001 |
| 0.6\|1 | 3.426 | 0.526 | 6.513 | <0.001 |

**Table S6.** (inverted) ANOVA model summaries for interaction strength test.

ANOVA - ALB

|  | Df | Sum Sq | Mean Sq | F value | p-value |
| --- | --- | --- | --- | --- | --- |
| Trophic interaction strength | 5 | 0.58 | 0.11642 | 4.098 | 0.001 |
| Residuals | 1638 | 46.53 | 0.02841 |  |  |

ANOVA - SCH

|  | Df | Sum Sq | Mean Sq | F value | p-value |
| --- | --- | --- | --- | --- | --- |
| Trophic interaction strength | 3 | 0.022 | 0.00722 | 0.18 | 0.91 |
| Residuals | 225 | 9.020 | 0.04009 |  |  |

**Figure S7.** The mean difference for each pair of trophic interaction strength level (“troph”) for region ALB after a Tukey HSD test.

**References**

Armas, C., Ordiales, R. and Pugnaire, F.I. (2004) ‘Measuring plant interactions: a new comparative index’, *Ecology*, 85(10), pp. 2682–2686. Available at: <https://doi.org/10.1890/03-0650>.

Ebert, G. (ed.) (2005) *Die Schmetterlinge Baden-Württembergs. 10: Ergänzungsband*. Stuttgart: Ulmer (Grundlagenwerke).

Griffith, D.M., Veech, J.A. and Marsh, C.J. (2016) ‘cooccur: Probabilistic species co-occurrence analysis in *R*’, *Journal of Statistical Software*, 69(Code Snippet 2). Available at: <https://doi.org/10.18637/jss.v069.c02>.

Richert, A. and Brauner, O. (2018) ‘Nektarpflanzen und andere Nahrungs- quellen sowie Raupennahrungspflanzen der Tagfalter von Brandenburg und Berlin (Lepidoptera: Rhopalocera et Hesperiidae)’, p. 87.

Veech, J.A. (2013) ‘A probabilistic model for analysing species co-occurrence’, *Global Ecology and Biogeography*, 22(2), pp. 252–260. Available at: <https://doi.org/10.1111/j.1466-8238.2012.00789.x>.

Veech, J.A. (2014) ‘The pairwise approach to analysing species co-occurrence’, *Journal of Biogeography*. Edited by M. Araújo, 41(6), pp. 1029–1035. Available at: <https://doi.org/10.1111/jbi.12318>.
